# PPARγ Controls Dectin-1 Expression Required for Host Antifungal Defense against *Candida albicans*

**DOI:** 10.1371/journal.ppat.1000714

**Published:** 2010-01-08

**Authors:** Amandine Galès, Annabelle Conduché, José Bernad, Lise Lefevre, David Olagnier, Maryse Béraud, Guillaume Martin-Blondel, Marie-Denise Linas, Johan Auwerx, Agnès Coste, Bernard Pipy

**Affiliations:** 1 UMR-MD3 EA2405 Université de Toulouse III; UPS; Polarisation des Macrophages et Récepteurs Nucléaires dans les Pathologies Inflammatoires et Infectieuses, PMRNP2I, Toulouse, France; 2 UMR-MD3; RH2PT Université de la Méditerranée - Ministère de la Défense, Marseille, France; 3 Institut de Génétique et de Biologie Moléculaire et Cellulaire, CNRS/INSERM/Université Louis Pasteur, Illkirch, France; 4 Institut Clinique de la Souris, Génopole Strasbourg, Illkirch, France; David Geffen School of Medicine at University of California Los Angeles, United States of America

## Abstract

We recently showed that IL-13 or peroxisome proliferator activated receptor γ (PPARγ) ligands attenuate *Candida albicans* colonization of the gastrointestinal tract. Here, using a macrophage-specific Dectin-1 deficient mice model, we demonstrate that Dectin-1 is essential to control fungal gastrointestinal infection by PPARγ ligands. We also show that the phagocytosis of yeast and the release of reactive oxygen intermediates in response to *Candida albicans* challenge are impaired in macrophages from Dectin-1 deficient mice treated with PPARγ ligands or IL-13. Although the Mannose Receptor is not sufficient to trigger antifungal functions during the alternative activation of macrophages, our data establish the involvement of the Mannose Receptor in the initial recognition of non-opsonized *Candida albicans* by macrophages. We also demonstrate for the first time that the modulation of Dectin-1 expression by IL-13 involves the PPARγ signaling pathway. These findings are consistent with a crucial role for PPARγ in the alternative activation of macrophages by Th2 cytokines. Altogether these data suggest that PPARγ ligands may be of therapeutic value in esophageal and gastrointestinal candidiasis in patients severely immunocompromised or with metabolic diseases in whom the prevalence of candidiasis is considerable.

## Author Summary

Since the early 1980s, *Candida albicans* has emerged as major cause of human disease, especially among immunocompromised individuals and those with metabolic dysfunction. The main host defense mechanisms against this yeast are engulfment and the production of reactive oxygen molecules by macrophages through Dectin-1 and the Mannose Receptor, two macrophage receptors for *Candida albicans* cell wall sugars. However, the contribution of these two receptors remains unclear. In our animal experiments, the lack of Dectin-1 in macrophages renders the animals more susceptible to gastrointestinal infection with *Candida albicans*, demonstrating the essential role of Dectin-1 in antifungal defense. In addition, our experiments established that the interaction between Dectin-1 and Mannose Receptor is important to orchestrate the host antifungal defense. Thus, *Candida albicans* clearance would be improved by Dectin-1 and Mannose Receptor up-regulation. Interestingly, we had established that the expression of these two receptors was increased by IL-13 through the activation of the nuclear receptor PPARγ, suggesting that PPARγ could be a therapeutic target to eliminate fungal infection. This paper, which highlights a new area of application of PPARγ ligands in infectious diseases, hence heralds the emergence of a new therapeutic strategy against fungal infection in severely immunocompromised patients or those with metabolic diseases.

## Introduction

Innate immunity is a conserved mechanism of host defense and is responsible for immediately recognizing microbial invasion through the engagement of pattern-recognition receptors (PRRs). These PRRs can recognize highly conserved microbial structures, known as pathogen-associated molecular patterns (PAMPs). The PRR ligands comprise carbohydrate structures, peptidoglycans or lipopolysaccharides. The best characterized family of PRRs is the Toll-like receptors (TLRs) originally supposed to mediate cellular signaling, but the membrane-associated C-type lectin receptors have since emerged as major receptors in functions related to pathogen binding, uptake, and killing. They also contribute to the initiation and the modulation of the immune response. The C-type lectins form a group of proteins with at least one lectin-like carbohydrate-recognition domain (CRD) in their extracellular carboxy-terminal domains [1]. The C-type lectin Dectin-1 is a major β-glucan receptor on the surface of macrophages, DCs, neutrophils and it is also expressed on certain lymphocytes [2]. This type II transmembrane receptor consists of a single CRD involved in the calcium-independent recognition of β-1, 3-glucans exposed on particles such as zymosan, or many fungal species, including *Saccharomyces, Pneumocystis, Aspergillus* and *Candida* [3]–[5].

*C.albicans* is the most common cause of opportunistic mycotic infections in severely immunocompromised hosts and during metabolic disease [6]. The cell wall of this yeast is almost exclusively composed of glycans, such as mannans and β-glucans [7]. Mannans are the major component of outer cell wall while β-(1,3)- and β-(1,6)-glucans are more prominent in the inner layer. However, there is some surface exposure of β-glucans, particularly in areas where yeast cells bud during mother–daughter cell separation [8],[9]. The recognition of the multilayered carbohydrate structures of the fungal cell wall depends on various PRRs, such as the Mannose Receptor (MR), and the β-glucan receptor Dectin-1 [9],[10]. The respective roles of these PPRs in the non-opsonic recognition of *C. albicans* by macrophages remain unclear. Several studies support the view that the MR plays a crucial role in non-opsonized *C.albicans* recognition and phagocytosis [9],[11],[12]. This receptor has also been shown to be associated with the production of proinflammatory cytokines and reactive oxygen species [9],[13]. Recently, the β-glucan receptor Dectin-1 was found to be the main non-opsonic receptor involved in fungal uptake [14]. In addition, Dectin-1-induced-signaling leads to the production of cytokines and non-opsonic phagocytosis of yeast by murine macrophages [15],[16]. Dectin-1 also mediates respiratory burst [17] and its involvement has been suggested in the activation and regulation of phospholipase A2 (PLA2) and cyclooxygenase-2 (COX-2) [18]. Dectin-1 signaling pathway activation depends on its cytoplasmic immunoreceptor tyrosine-based activation motif (ITAM) the phosphorylation of which by Src kinase leads to the recruitment of spleen tyrosine kinase Syk in macrophages [19]. Although the contribution of the MR and Dectin-1 in non-opsonized *C.albicans* recognition, phagocytosis and killing is established, the point of intervention of these receptors in these processes remains unclear. In addition, depending on the context of the macrophage activation, the expression profile of PRRs is different. Thus, the change in the PRRs expression has to be taken into account in studying the involvement of these receptors in antifungal functions.

In mice, the expression of Dectin-1 can be influenced by various cytokines, steroids and microbial stimuli. Interleukin-4 (IL-4) and IL-13, for example, which are associated with the alternative activation of macrophages (macrophages M2), markedly increase the expression of Dectin-1 at the cell surface, whereas LPS and dexamethasone repress Dectin-1 expression [20]. Nevertheless, the Dectin-1 regulation pathway remains unclear. Empirical data suggest that the increase in Dectin-1 expression by IL-4 involved the STAT signaling pathway [21]. Moreover, another study showed in genetic models of macrophage specific Peroxisome proliferator-activated receptor γ (PPARγ) or STAT-6 knockout mice, that the IL-4/IL-13/STAT-6/PPARγ axis is required for the maturation of alternatively activated macrophages [22]. Therefore, in this context, the signaling pathway involved in the modulation of Dectin-1 expression remains to be elucidated.

In this study we determined the respective roles of the MR and Dectin-1 in the control of fungal infection. Interestingly, we showed that *in vitro* and *in vivo*, Dectin-1 is essential both to trigger the phagocytosis of non-opsonized *Candida albicans* and the respiratory burst after yeast challenge and to control fungal gastrointestinal infection. These data also established that the MR alone is not sufficient to trigger antifungal functions during macrophage alternative activation, indicating a cooperative role for Dectin-1 and the MR in the induction of the host antifungal response against *Candida albicans*. Moreover, we showed for the first time the involvement of the PPARγ signaling pathway in the regulation of Dectin-1 expression by IL-13. This report highlights that PPARγ ligands could be of therapeutic benefit in the resolution of fungal infections in patients severely immunocompromised or with metabolic diseases in whom the prevalence of candidiasis is considerable.

## Results

### Involvement of Dectin-1 and the Mannose Receptor in the antifungal functions of alternatively activated macrophages

**Figure 1:**
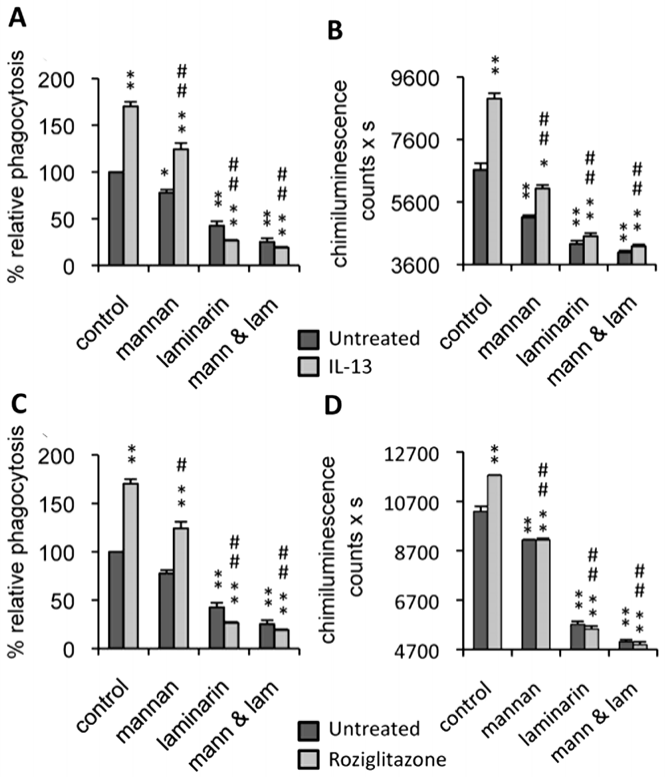
Dectin-1 and the Mannose Receptor are implicated in antifungal functions of macrophages treated with IL-13 or PPARγ ligand. Peritoneal macrophages were cultured with IL-13 (50 ng/mL) (A and B) or rosiglitazone (5 µM) (C and D). Mannan (mann) and/or soluble β-glucan (laminarin, lam) solutions were incubated at 4°C for 20 min until the phagocytosis and respiratory burst experiments. (A and C) The phagocytosis of non-opsonized *C.albicans* (ratio 1∶6) by macrophages was measured at 37°C after exposure to FITC-labeled *C.albicans* for 60 min. The amount of fluorescence was determined using a FACS based approach. The distinction between internalized yeast cells and those attached to macrophage surface was done *via* quenching the FITC-fluorescence with trypan blue. Data are expressed as percentage relative to untreated control macrophages and are means±SE of three separate experiments. (B and D) Non-opsonized *C.albicans*-induced respiratory burst of macrophages (ratio 1∶3) was measured by chimiluminescence. Total chemiluminescence emission (area under the curve expressed in counts x seconds) was observed continuously for 60 min in the presence or absence of non-opsonized *C. albicans*. The data are the means±SE of three separate experiments. ** (p<0.01) and * (p<0.05) indicates a significant difference compared with the untreated macrophages. ## (p<0.01) and # (p<0.05) indicates a significant difference compared with the treated control macrophages.

To explore the role of Dectin-1 and the MR in the control of fungal infection by alternatively activated macrophages (M2), we studied the phagocytosis of non-opsonized *C.albicans* and the production of yeast-induced reactive oxygen species (ROS) by macrophages, in the presence or absence of soluble receptor blocking agents (laminarin and mannan). The phagocytosis of non-opsonized *C.albicans* was significantly increased by IL-13 treatment (Figure 1A). This enhancement of phagocytosis in M2 activation was reduced by mannan. Moreover, laminarin or the association of mannan and laminarin blocked yeast internalization (Figure 1A).

Since *C.albicans* stimulates phagocytosis, and since this function contributes to the triggering of ROS production, we examined the respiratory burst induced by non-opsonized *C.albicans* in IL-13 polarized macrophages. As in the phagocytosis experiment, ROS production was enhanced by IL-13, and clearly reduced by the addition of soluble mannan and/or laminarin (Figure 1B).

Consistent with the critical role of PPARγ activation in the maturation of alternatively activated macrophages, we explored these two antifungal functions in PPARγ ligand-primed macrophages. Interestingly, these functions were enhanced by rosiglitazone, a PPARγ specific ligand, and decreased by mannan and/or laminarin pretreatment, as observed during alternative activation by IL-13 (Figure 1C, 1D). Altogether, these data showed that the antifungal functions of macrophages promoted by IL-13 or a PPARγ ligand involved Dectin-1 and the MR.

### Phenotypic and functional characterizations of macrophages from macrophage-specific Dectin-1 deficient mice

**Figure 2:**
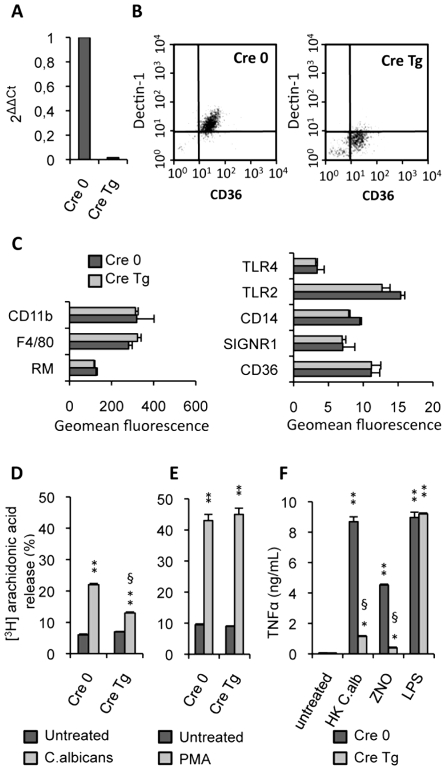
Characterization of Dectin-1 conditional knock-out macrophages. (A) The Dectin-1 mRNA level of Dectin-1 control (Cre 0) and Dectin-1 knockout (Cre Tg) peritoneal macrophages was quantified by quantitative real-time RT-PCR. Values are representative of data obtained from three mice. (B) The surface protein level of Dectin-1 was measured by flow cytometry on the Dectin-1 control (Cre 0) and Dectin-1 knockout (Cre Tg) peritoneal macrophages. Plots are representative of data obtained from six mice. (C) The protein level of several receptors (CD11b, Mannose Receptor MR, TLR4, TLR2, SIGNR1, CD36) and markers (F4/80 and CD14) on the macrophage surface was determined by flow cytometry on the Dectin-1 control (Cre 0) and Dectin-1 knockout (Cre Tg) peritoneal macrophages. (D and E) Released [3H]arachidonic acid is expressed as the percentage of [3H]arachidonic acid in the culture medium divided by the total incorporated [3H]arachidonic acid in murine peritoneal macrophages. The release of [3H]arachidonic acid was determined after incubation for 120 min of peritoneal Dectin-1-control and Dectin-1-knockout macrophages with non-opsonized *C.albicans* (ratio 1∶3) or PMA (100 nM). The data represent the means±SE of three separate experiments. (F) TNFα production by Dectin-1 control (Cre 0) and Dectin-1 knockout (Cre Tg) macrophages after 24 h of stimulation with heat-killed *C.albicans* (ratio 1∶3), ZNO (2 µg/mL) or LPS (100 ng/mL). Data are the means±SE of three separate experiments. ** (p<0.01) and * (p<0.05) indicates a significant difference compared with the respective Cre 0 or Cre Tg control. § (p<0.05) indicates a significant difference between Cre 0 and Cre Tg.

To unequivocally determine the role of Dectin-1 in *C.albicans* elimination, we generated Dectin-1 receptor conditional knockout mice, in which Dectin-1 was selectively disrupted in phagocytic cells. First, we generated mice that carried conditional *Dectin-1* alleles (*Dectin-1* ^L2/L2^ mice). To generate spatially controlled mouse mutants for the *Dectin-1* gene in the macrophages, mice carrying the floxed *Dectin-1* L2 alleles were crossed with transgenic mice that expressed the Cre recombinase under the control of the mouse lysozyme M promoter. Quantitative real-time RT-PCR and flow cytometry confirmed that the mRNA and protein levels of Dectin-1 were abrogated in phagocyte cells (Figure 2A, 2B).

To explore the antigen phenotype of the Dectin-1 knockout macrophages, we studied the protein level of several receptors and markers on the macrophage surface. No significant changes in protein levels were detected between the control (Cre 0) and Dectin-1 knockout (Cre Tg) macrophages (Figure 2C) showing that in Dectin-1 knockout macrophages there was no compensatory increase in the expression of other PRRs.

It had previously been shown that arachidonic acid release induced by *C.albicans* was inhibited by preincubation with soluble glucan phosphate [18]. In this context, to characterize the functional capacity of Dectin-1 knockout macrophages, we investigated the ability of non-opsonized *C.albicans* to stimulate arachidonic acid release by the control (Cre 0) or Dectin-1 knockout (Cre Tg) peritoneal macrophages. *C.albicans* challenge reduced release of arachidonic acid by Cre Tg macrophages but not by Cre 0 macrophages (Figure 2D). Interestingly, Cre Tg macrophages were able to release arachidonic acid in response to phorbol myristate acetate (PMA) known to mediate arachidonic acid release *via* surface receptor independent pathway (Figure 2E). We confirmed that Cre Tg macrophages had no abnormalities in their lipid metabolism and that Dectin-1 was required for arachidonic acid metabolism in response to *C.albicans*.

We then investigated the ability of these Dectin-1 knockout macrophages to produce inflammatory cytokines. TNFα production by control (Cre 0) and Dectin-1 knockout (Cre Tg) macrophages was evaluated in response to heat-killed *C.albicans*. The Dectin-1 knockout macrophages failed to produce TNFα in response to heat-killed *C.albicans* or non-opsonized zymosan (ZNO) (Figure 2F). However, LPS stimulation demonstrated that Dectin-1 knockout macrophages do not have a generalized defect in TNFα production (Figure 2F).

All these data showed that this mutation did not affect the phenotype and the Dectin-1 independent functional capacities of the macrophages and hence provide an appropriate model to explore the involvement of Dectin-1 in *C.albicans* clearance during alternative activation.

### The Mannose Receptor and Dectin-1 are involved in the elimination of non-opsonized *C.albicans* at different stages of clearance

We investigated the effect of the macrophage-specific Dectin-1 deletion both on non-opsonized *C.albicans* recognition and on the antifungal functions in a M2 activation context.

**Figure 3:**
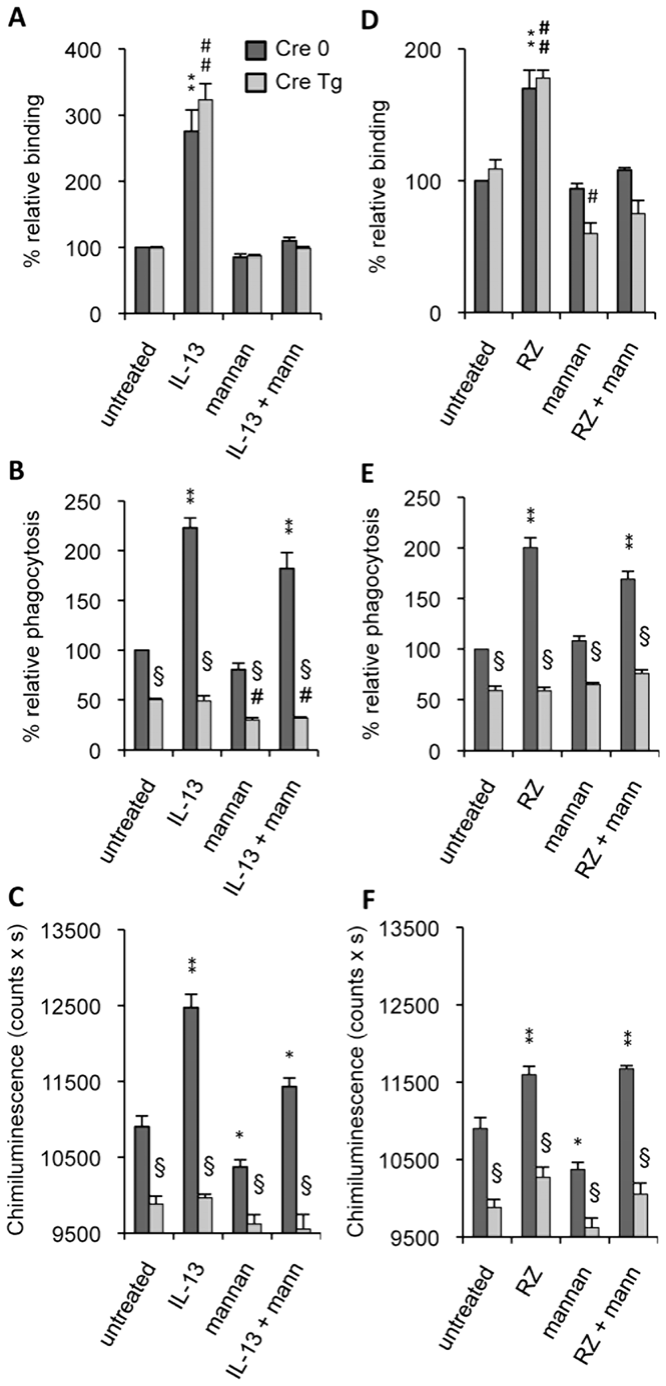
Dectin-1 and the Mannose Receptor are required in different stages of *C.albicans* clearance. Dectin-1 control (Cre 0) and Dectin-1 knockout (Cre Tg) peritoneal macrophages were cultured with IL-13 (50 ng/mL) (A–C) or with rosiglitazone (RZ) (5 µM) (D–F). Mannan (mann) solution was incubated at 4°C for 20 min until the binding, phagocytosis and respiratory burst experiments. (A and D) The binding experiment of non-opsonized *C.albicans* by macrophages was measured at 4°C after challenge with FITC-labeled *C.albicans* for 20 min (ratio 1∶6). The amount of fluorescence was determined using a FACS based approach. Data are expressed as the percentage relative to the untreated Dectin-1 control (Cre 0) macrophages and are the means±SE of three separate experiments. (B and E) The phagocytosis of non-opsonized *C.albicans* by macrophages was measured at 37°C after challenge with FITC-labeled *C.albicans* for 60 min (ratio 1∶6). The amount of fluorescence was determined using a FACS based approach. The distinction between internalized yeast cells and those attached to macrophage surface was done *via* quenching the FITC-fluorescence with trypan blue. Data are expressed as the percentage relative to the untreated Dectin-1 control (Cre 0) macrophages and are the means±SE of three separate experiments. (C and F) The respiratory burst of the Dectin-1 control (Cre 0) and Dectin-1 knockout (Cre Tg) macrophages induced by non-opsonized *C.albicans* was measured by chimiluminescence (ratio 1∶3). Total chemiluminescence emission (area under the curve expressed in counts x seconds) was observed continuously for 60 min. Data are the means±SE of three separate experiments. ** (p<0.01) and * (p<0.05) indicates a significant difference compared with the Cre 0 untreated macrophages. ## (p<0.01) indicates a significant difference compared with the Cre Tg untreated macrophages. § (p<0.05) indicates a significant difference between Cre 0 and Cre Tg.

We first looked at the binding of non-opsonized *C.albicans* on resident peritoneal macrophages. Untreated Dectin-1-control (Cre 0) and Dectin-1 knockout (Cre Tg) macrophages bound non-opsonized yeast at 4°C (Figure 3A). Interestingly, M2 polarization of macrophages by IL-13 strongly enhanced the binding of non-opsonized *C.albicans* equally in Cre 0 and Cre Tg macrophages, showing that Dectin-1 is not required for the initial binding of the yeast to untreated as well as to alternatively activated macrophages. Then to explore the role of the MR in binding non-opsonized *C.albicans*, we pretreated Cre 0 and Cre Tg macrophages with soluble mannan (Figure 3A). The addition of mannan did influence slightly the binding of non-opsonized *C.albicans* to resident macrophages, suggesting that the MR and other PRRs were implicated in this stage of recognition. Interestingly, the binding by alternatively activated macrophages was strongly inhibited by mannan. We concluded that the MR is the main PRR for the initial binding in M2 activation.

To further investigate the respective roles of the MR and Dectin-1 in antifungal functions we studied the ability of Dectin-1 control (Cre 0) and Dectin-1 knockout (Cre Tg) macrophages to engulf *C.albicans* and to produce reactive oxygen species in the presence of mannan. Non-opsonized *C.albicans* phagocytosis was decreased in Cre Tg resident macrophages (Figure 3B). IL-13 increased yeast internalization in Cre 0 macrophages, but importantly, it failed to improve the antifungal response in Cre Tg macrophages. The addition of mannan slightly changed the uptake of non-opsonized *C.albicans* by resident and M2 polarized macrophages. Together these data showed that Dectin-1 was essential for triggering the phagocytosis of non-opsonized *C.albicans* both in resident and in alternatively activated macrophages.

Consistent with the phagocytosis results, the reactive oxygen species production induced by non-opsonized yeast was increased by IL-13 only in Cre 0 macrophages. Moreover, the addition of mannan slightly decreased ROS production. These results showed that Dectin-1 is the main receptor involved in this antifungal function (Figure 3C) and that Dectin-1 is very important in triggering the phagocytosis of yeast and the *C.albicans*-induced respiratory burst in alternatively activated macrophages.

To assess the involvement of PPARγ in the MR- and Dectin-1-dependent antifungal functions, we studied the binding and phagocytosis of *C.albicans*, and ROS production in the presence of rosiglitazone, a specific PPARγ-ligand. Rosiglitazone strongly increased the binding of non-opsonized *C.albicans* by Cre 0 and Cre Tg macrophages but failed to trigger phagocytosis and ROS production by Cre Tg macrophages (Figure 3D–F). Moreover, the addition of mannan only affected the binding. These results suggest the involvement of PPARγ in the mechanisms of response to non-opsonized *C.albicans* dependent on the MR and/or Dectin-1 receptors during M2 activation by IL-13.

### PPARγ is involved in the regulation of Dectin-1 expression in alternatively activated macrophages

**Figure 4:**
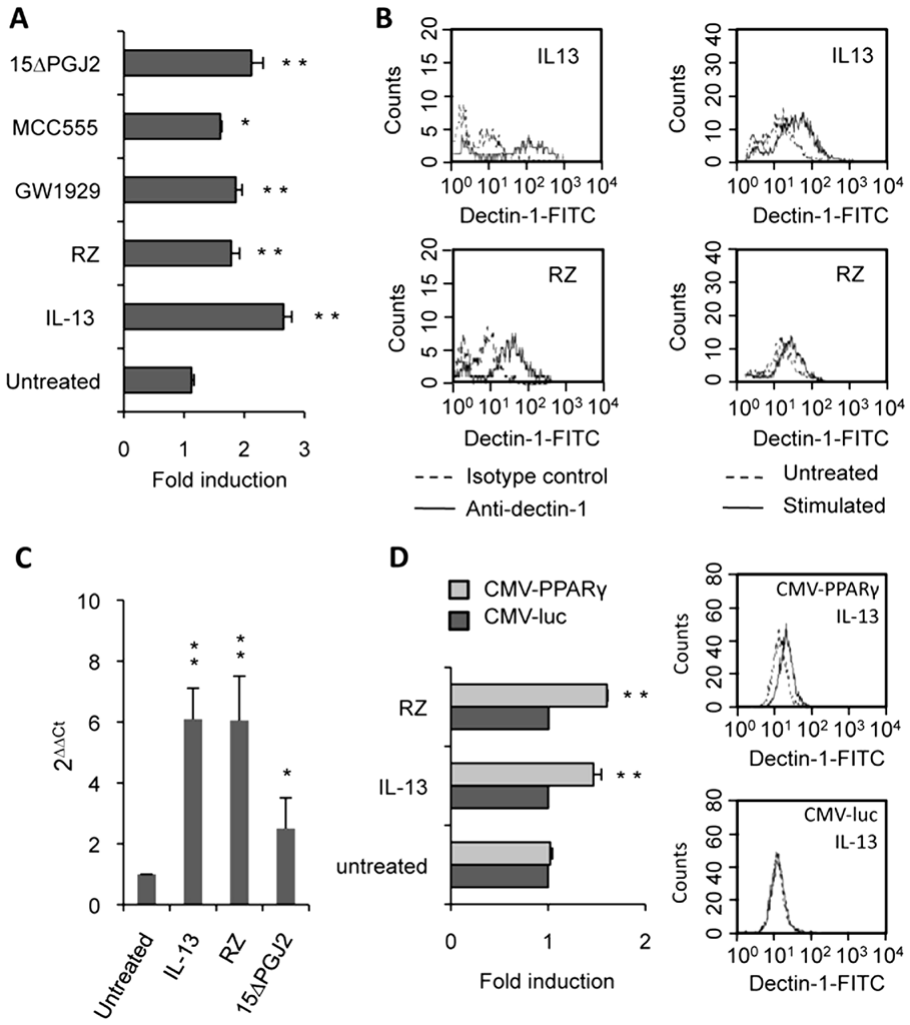
Dectin-1 expression depends on PPARγ activation by IL-13 or PPARγ-specific ligands. (A) The surface protein level of Dectin-1 on peritoneal macrophages was measured by flow cytometry after treatment with IL-13 (50 ng/mL), rosiglitazone (RZ) (5 µM), 15d-PGJ2 (1 µM), MCC555 (5 µM) or GW1929 (1 µM). The changes in Dectin-1 induction were normalized to the untreated control value. Data are the means±SE of three separate experiments. (B) Representative FACS profiles of Dectin-1 (filled histograms) and isotype control labeling (unfilled histograms) in treated macrophages. Representative Dectin-1 FACS profiles of untreated macrophages (unfilled histograms) and treated (filled histograms) macrophages. (C) The mRNA level of Dectin-1 on peritoneal macrophages was quantified by quantitative real-time RT-PCR after treatment with IL-13 (50 ng/mL), rosiglitazone (RZ) (5 µM) or 15d-PGJ2 (1 µM). Data are the means±SE of three separate experiments. ** (p<0.01) and * (p<0.05) indicates a significant difference compared with the untreated macrophages. (D) The protein level of Dectin-1 on the murine cell line RAW264.7 transiently transfected with pCMV-luciferase (CMV-luc) or with pCMV-mPPARγ (CMV-PPARγ) and after treatment with IL-13 or rosiglitazone (RZ). Representative Dectin-1 FACS profiles of untreated (unfilled histograms) and treated (filled histograms) macrophages were obtained by flow cytometry. The changes in Dectin-1 induction were normalized to the untreated RAW 264.7 cells transfected with pCMV-luc. Data are the means±SE of three separate experiments. ** (p<0.01) and * (p<0.05) indicates a significant difference compared with the untreated RAW 264.7 cells transfected with pCMV-luc.

In this context of M2 polarization, we explored the involvement of the PPARγ pathway in the regulation of Dectin-1 expression. We stimulated resident peritoneal macrophages with synthetic (Rosiglitazone, MCC555 and GW1929), natural (15ΔPGJ2) PPARγ-ligands or IL-13. FACS profiles showed that the Dectin-1 protein level at the surface of the macrophages was markedly up-regulated by IL-13 and PPARγ-specific ligands (Figure 4A, 4B). Equally, Dectin-1 mRNA expression was significantly enhanced by IL-13, Rosiglitazone and 15ΔPGJ2 (Figure 4C). To assess the involvement of PPARγ in the modulation of Dectin-1 expression, a PPARγ-deficient cell line RAW264.7 [23] was transiently transfected with the pCMV-mPPARγ expression vector. After IL-13 or Rosiglitazone treatment, the level of Dectin-1 protein expression was higher in cells transfected with the pCMV-mPPARγ than in control cells transfected with the pCMV-luc vector (Figure 4D). This result suggests that the induction of Dectin-1 by IL-13 is PPARγ-dependent.

**Figure 5:**
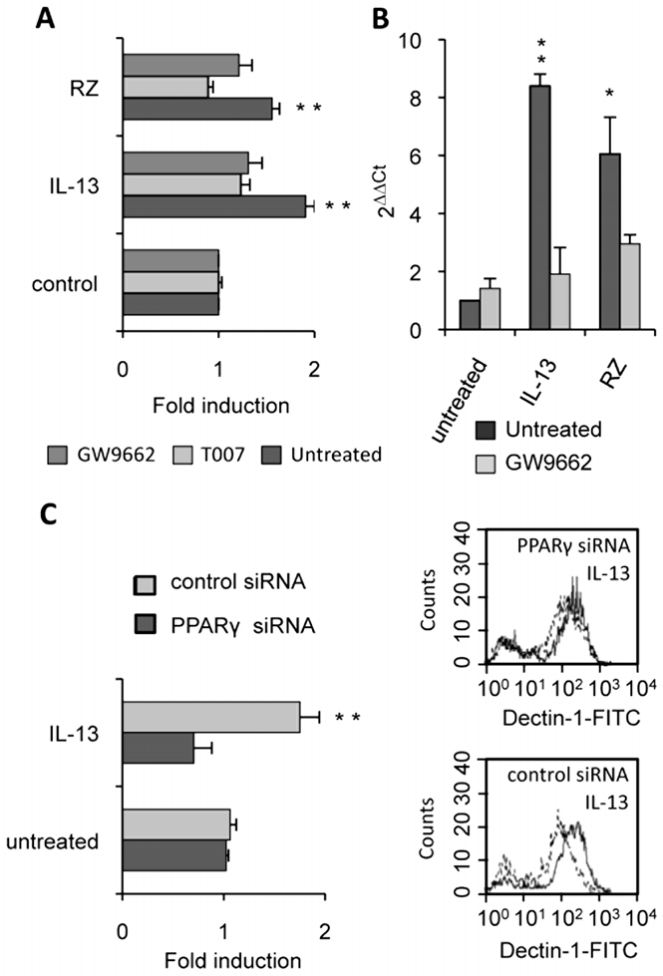
PPARγ inhibition in M2 polarized macrophages abolishes the increase of Dectin-1. (A) The protein level of Dectin-1 on peritoneal macrophages was measured by flow cytometry after treatment with IL-13 (50 ng/mL) or rosiglitazone (RZ) (5 µM) in the presence of the PPARγ antagonists (GW9662 (5 µM) and T007 (2 µM)). Data are the means±SE of three separate experiments. (B) Dectin-1 mRNA level of peritoneal macrophages was quantified by quantitative real-time RT-PCR after treatment with IL-13 (50 ng/mL) or rosiglitazone (RZ) (5 µM) in the presence of the PPARγ antagonist (GW9662 (5 µM)). Data are the means±SE of three separate experiments. ** (p<0.01) and * (p<0.05) indicates a significant difference compared with the untreated macrophages. (C) The surface protein level of Dectin-1 on peritoneal macrophages transfected with siRNA targeting PPARγ (PPARγ siRNA) or control siRNA (control siRNA) and stimulated by IL-13. Representative Dectin-1 FACS profiles of untreated (unfilled histograms) and treated (filled histograms) macrophages were obtained by flow cytometry. The changes in Dectin-1 receptor levels were normalized to the untreated macrophages transfected with the siRNA control. Data are the means±SE of three separate experiments. ** (p<0.01) and * (p<0.05) indicates significant difference compared with the untreated macrophages transfected with the siRNA control.

To unequivocally prove the involvement of the PPARγ-pathway, we blocked PPARγ activation with two irreversible PPARγ antagonists (T007 and GW9662) or with a specific PPARγ siRNA. Macrophages treated with the antagonists failed to up-regulate Dectin-1 expression after exposure to IL-13 and PPARγ-specific ligands, as shown by FACS analysis or quantitative real-time RT-PCR (Figure 5A, 5B). Moreover, we demonstrated that silencing PPARγ expression in macrophages with siRNA also abolished the specific increase of Dectin-1 by IL-13 (Figure 5C).

All these data together prove that the nuclear receptor PPARγ is required for the induction of Dectin-1 by IL-13 in mouse peritoneal macrophages.

### Cytosolic PLA2 contributes to the induction of Dectin-1 by IL-13

**Figure 6:**
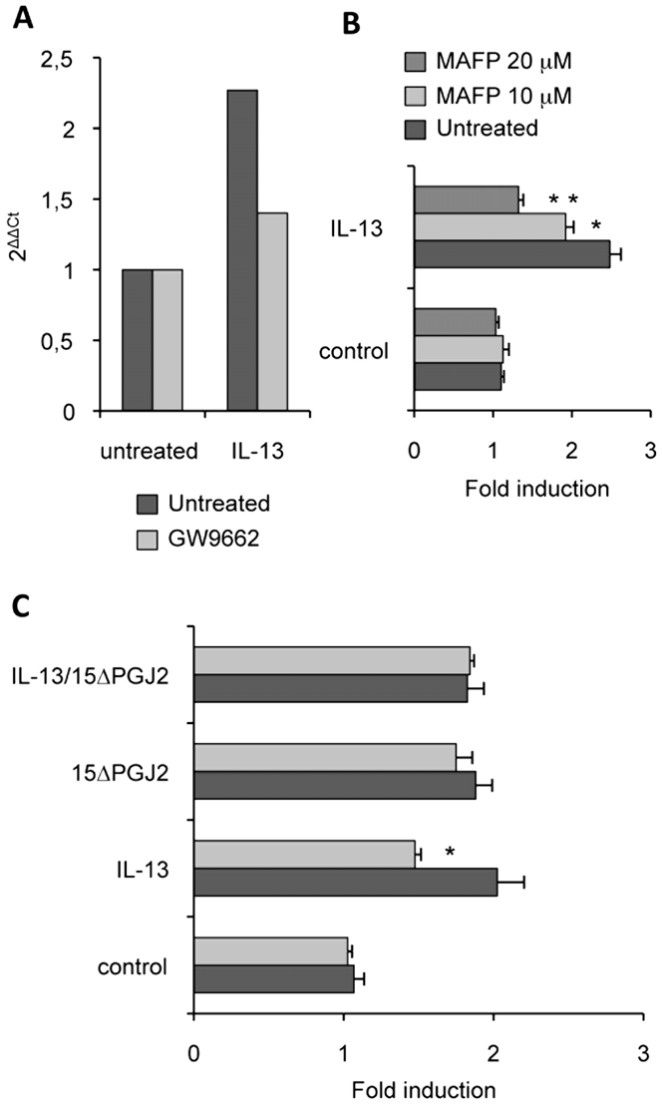
cPLA2 is involved in Dectin-1 induction by IL-13. (A) The Dectin-1 mRNA level of peritoneal macrophages was measured by quantitative real-time RT-PCR after treatment of peritoneal macrophages by an irreversible cPLA2 antagonist (MAFP) and by IL-13. Data are the means±SE of three separate experiments. (B) The protein level of Dectin-1 was measured by flow cytometry on peritoneal macrophages after treatment with MAFP (10 µM and 20 µM) and with IL-13 (50 ng/mL). Data are the means±SE of three separate experiments. (C) The protein level of Dectin-1 was measured by flow cytometry on peritoneal macrophages after treatment with MAFP and IL-13 and/or 15d-PGJ2 (1 µM). Data are the means±SE of three separate experiments. ** (p<0.01) and * (p<0.05) indicates a significant difference compared with the IL-13-treated macrophages.

Because cPLA2 regulates the synthesis of 15ΔPGJ2, an endogenous PPARγ-ligand, we studied the effects of a specific cPLA2 inhibitor (MAFP) on Dectin-1 expression. We showed that MAFP inhibited macrophage Dectin-1 mRNA expression (Figure 6A). In line, the level of Dectin-1 protein was decreased in a dose-dependent manner by the treatment of macrophages with MAFP (Figure 6B).The addition of 15ΔPGJ2 restored the induction of Dectin-1 by IL-13 (Figure 6C). Thus, IL-13 regulates Dectin-1 expression by controlling the production of the PPARγ endogenous ligand through cPLA2 activation.

### Dectin-1 is required to control *C.albicans* gastrointestinal infection *in vivo*

**Figure 7:**
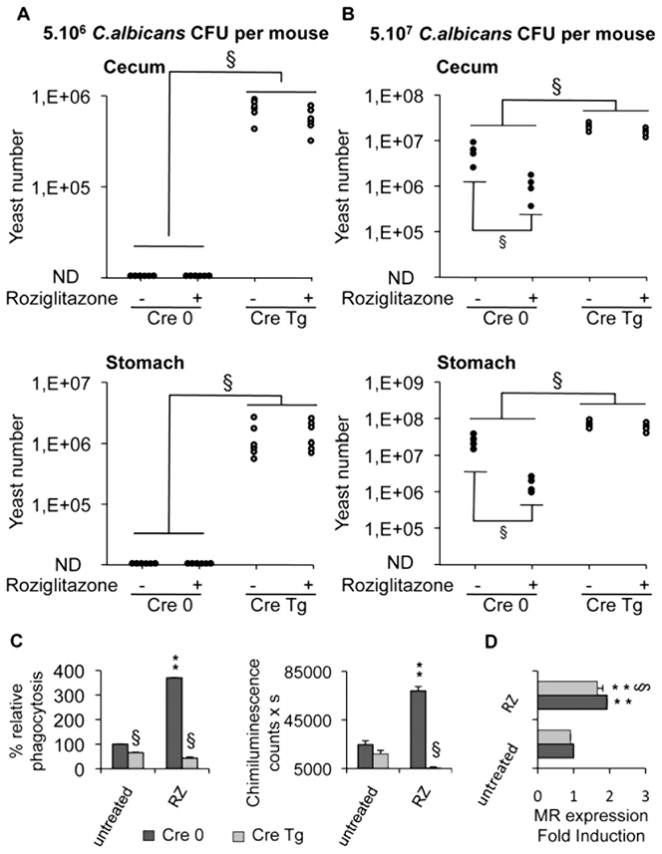
Dectin-1-knockout mice are more susceptible than Dectin-1-wildtype mice to *C. albicans* gastrointestinal infection. (A and B) Quantification of *C. albicans* fungal burden in the gastrointestinal tract (stomach and cecum) of Dectin-1-control mice Cre 0 (filled circles) and Dectin-1-knockout mice Cre Tg (open circles) at 5 day after oral infection with 5.10^6^ CFU (A, n = 6) or with 5.10^7^ CFU (B, n = 4) in standard conditions or after treatment with rosiglitazone (RZ) (2.8 µg/g of mouse). Each symbol represents an individual mouse. § (p<0.05) indicates a significant difference between group of mice. (C) Phagocytosis and ROS production were measured on peritoneal macrophages from Dectin-1 knockout (Cre Tg) mice at 5 day after oral infection with 5.10^6^ CFU in standard conditions or after treatment with rosiglitazone (RZ). The phagocytosis of non-opsonized *C.albicans* by macrophages was measured at 37°C after exposure to FITC-labeled *C.albicans* for 60 min (ratio 1∶6). The amount of fluorescence was determined using a FACS based approach. The distinction between internalized yeast cells and those attached to macrophage surface was done *via* quenching the FITC-fluorescence with trypan blue. Data are expressed as the percentage relative to untreated Dectin-1 control (Cre 0) macrophages and are the means±SE (n = 6). The respiratory burst of macrophages induced by non-opsonized zymosan (ZNO) (2 µg/mL) was measured by chimiluminescence. Total chemiluminescence emission (area under the curve expressed in counts x seconds) was observed continuously for 60 min. Data are the means±SE (n = 6). ** (p<0.01) indicates a significant difference compared with the Cre 0 untreated macrophages. § (p<0.05) indicates a significant difference between Cre 0 and Cre Tg. (D) The MR surface protein level was measured by flow cytometry on peritoneal macrophages from Dectin-1 control (Cre 0) or Dectin-1 knockout (Cre Tg) mice at day 5 after oral infection with 5.10^7^ CFU in standard conditions or after treatment with rosiglitazone (RZ). Data are the means±SE (n = 4). ** (p<0.01) indicates a significant difference compared with the Cre 0 control. § (p<0.05) indicates a significant difference between Cre 0 and Cre Tg.

To assess precisely the involvement of Dectin-1 in the development of gastrointestinal candidiasis and in the antifungal effect of PPARγ ligands, we studied the susceptibility to *Candida* infection of macrophage-specific Dectin-1 deficient (Cre Tg) mice treated or not with rosiglitazone. In macrophage-specific Dectin-1 deficient mice infected with 5.10^6^ *C.albicans* cells, the yeast extensively colonized the stomach and cecum whereas in control mice the colonization was undetectable (>10^4^) (Figure 7A). These results demonstrated that Dectin-1 plays an important role in host defense against gastrointestinal infection with *C.albicans*. Interestingly, treating mice with rosiglitazone did not improve the *Candida* clearance in the gastrointestinal tract, suggesting that rosiglitazone needs Dectin-1 to exert its antifungal effect. To ensure that the lack of the rosiglitazone effect was due to the absence of Dectin-1, we infected control and Dectin-1 deficient mice orally with a larger quantity of yeast (5.10^7^ *C.albicans*) and then we studied the effect of rosiglitazone on the outcome of this gastrointestinal infection (Figure 7B). In this gastrointestinal model of Candida infection, the yeast colonized the stomach and the cecum in both control and Dectin-1 deficient mice. In addition, the gastrointestinal colonization was considerably higher in the Dectin-1 deficient mice, confirming the major involvement of Dectin-1 in the host defense against *C. albicans*. As expected, rosiglitazone improved *Candida* clearance only in control mice, demonstrating that the lack of the rosiglitazone effect in Dectin-1 deficient mice was dependent on the lack of Dectin-1.

We then investigated the ability of macrophages from infected macrophage-specific Dectin-1 deficient mice to phagocytose yeast and to release reactive oxygen intermediates. In these two models of *Candida* gastrointestinal infection, we showed that Cre Tg macrophages failed to engulf *C. albicans* and to produce ROS (Figure 7C, data not shown). In addition, the treatment *in vivo* with rosiglitazone increased phagocytosis and the production of ROS in macrophages from Cre 0 mice, whereas this treatment did not increase these functions in Cre Tg macrophages. Nevertheless, in macrophages from Cre 0 or Cre Tg mice, the rosiglitazone treatment increased the expression of the MR (Figure 7D). Altogether these data demonstrated that in the absence of Dectin-1 the MR is not sufficient to trigger the antifungal functions and that the antifungal effect of rosiglitazone is Dectin-1 dependent.

## Discussion

*Candida albicans* causes significant and recurrent infections among immunocompromised hosts and during metabolic dysregulation. The interactions between hosts and fungal pathogens are mediated by mannans and β-glucans, the major cell wall components of *C.albicans*. The MR and Dectin-1 are the main pattern recognition receptors of the phagocytic system involved in *C.albicans* elimination.

Classically, the MR was described as mediating non-opsonized *C.albicans* recognition through the mannan chains on the outer yeast cell wall [9],[11],[12]. The MR is mainly involved in the uptake and phagocytosis of yeasts but it has also been shown to be involved in the production of TNFα, IL-1β, IL-6 and reactive oxygen species [9],[13] and to modulate the pro-inflammatory effects in collaboration with TLRs. However, other studies have shown the contribution of the β-glucan receptor Dectin-1 in response to non-opsonized *C.albicans*. Dectin-1 triggered the phagocytosis of the yeast cell wall particle zymosan by macrophages and dendritic cells [10]. It is now established that Dectin-1 mediates macrophage phagocytosis of *C.albicans* yeast [24] but not hyphae [8]. In addition, Dectin-1 also signals the release of reactive oxygen species, TNFα, IL-2, IL-6, IL-10 and IL-23 [3],[15],[25],[26]. Despite the fact that these two receptors are involved in common functions, a better understanding of the role of these receptors in the interaction between fungi and macrophages and in the antifungal functions of innate immune cells is necessary.

This study provides new data illustrating the relevant link between Dectin-1, the MR and the antifungal response during alternative activation of macrophages. We have demonstrated that depending on the context of macrophage activation, the receptors involved in yeast initial binding are different. Interestingly, we showed that the MR is the main PRR for the initial binding only in a M2 activation context in which the macrophages strongly expressed the MR at their surface. In contrast, the results of binding of non-opsonized *C.albicans* by resident macrophages showed that the MR is not involved in the recognition of non-opsonized *C.albicans*. This finding is consistent with the study which showed that resident macrophages do not express the MR [2]. In addition, we showed that Dectin-1 is not involved in the initial binding of non-opsonized *C.albicans* in resident macrophages. Moreover, in M2 activated macrophages, Dectin-1 is also not implicated in recognition of non-opsonized *C.albicans*. This finding is in line with a study on dendritic cells which showed that the addition of anti-MR and anti-DC-SIGN blocking agents inhibited the binding of non-opsonized *C.albicans* whereas the addition of an anti-Dectin-1 blocking agent did not change this recognition [27]. However, after 60 min of interaction between non-opsonized *C.albicans* and macrophages *in vitro*, we showed that Dectin-1 is sufficient to trigger the phagocytosis of non-opsonized *C.albicans* and respiratory burst after challenge with the yeast. Indeed, our results obtained with laminarin are consistent with our data using Dectin-1 knockout macrophages that confirm that the impairment of this receptor strongly decreased the antifungal response. Heinsbroek and coworkers have reported that the phagocytosis of unopsonized *C.albicans* by thioglycollate-ellicited macrophages of Dectin-1 deficient mice was reduced by 80%, showing that Dectin-1 is the main PRR for the initial phagocytosis by thioglycollate-ellicited macrophages [14]. These authors also have showed the lack of effects on MR in phagocytosis of *Candida* by thioglycollate-ellicited macrophages in which the MR is mainly intracellular in location. These data are also consistent with studies which showed that silencing Dectin-1 expression in macrophages with micro-RNA abolishes the zymosan-induced ROS production [25] and that the Dectin-1 engagement was sufficient to trigger phagocytosis and ROS production stimulated by zymosan [26] or by *C.albicans* [8]. In our study we demonstrated that the initial binding of the yeast through the MR does not seem to be directly involved in Dectin-1-dependant *C.albicans* uptake and respiratory burst. Altogether these results strongly suggest that another receptor could be implicated in the initial step of the Dectin-1-dependant phagocytosis of non-opsonized *C.albicans* and ROS production. This hypothesis is in line with a recent study which showed that complement receptor 3 accumulates at the site of particle binding and hence suggests it has a role during fungal recognition [14].

The major contribution of Dectin-1 in *C.albicans* internalization and ROS production *in vitro* supports our *in vivo* study which showed that Dectin-1 knockout mice were more susceptible to gastrointestinal candidiasis. This increased *Candida* colonization of the stomach and cecum in macrophage-specific Dectin-1 deficient mice correlated with the decrease in the effective functions of their macrophages *ex vivo*, as observed *in vitro*. These data demonstrated that Dectin-1 is required for the host defense to GI infection with *C.albicans* and support the role of Dectin-1 in the *in vivo* control of *C.albicans* infection [28]. We recently showed that i.p. treatment of immunocompetent and immunodeficient (RAG-2^−/*−*^) mice with natural and synthetic PPARγ-specific ligands or with IL-13 decreased *C. albicans* colonization of GI tract 8 days following oral infection with the yeast [13]. Similarly, we demonstrated here that rosiglitazone, a specific PPARγ ligand, improved GI fungal clearance only in control mice and this amelioration was correlated with an increased in antifungal functions of their macrophages *ex vivo* (*Candida* phagocytosis and ROS production). Nevertheless, the treatment of macrophage-specific Dectin-1 deficient mice with rosiglitazone did not enhance the *Candida* elimination in GI tract while the rosiglitazone treatment increased the expression of MR. Altogether these data established that the MR alone is not sufficient to trigger the antifungal functions and the antifungal action of rosiglitazone is dependent on Dectin-1. We also show that the phagocytosis of yeast and the release of reactive oxygen intermediates in response to *Candida albicans* challenge are impaired in macrophages from Dectin-1 deficient mice treated with rosiglitazone. This *in vivo* study demonstrates that Dectin-1 is essential both to trigger the phagocytosis of non-opsonised *C.albicans* and the respiratory burst after yeast challenge, and to control fungal GI infection. In parallel, the involvement of the MR in the initial binding during M2 activation demonstrates that the MR and Dectin-1 are essential for an optimal antifungal host defence. Altogether these data suggest a cooperative role for these two receptors in the induction of the immune response against *Candida* by rosiglitazone. This cooperation between the MR, Dectin-1 and TLR2 was also demonstrated in the pathway involved in IL-1β production by *C.albicans* [29].

The involvement of Dectin-1 in improving the resolution of candidiasis by PPARγ ligands is in line with our results which showed for the first time that the increase in Dectin-1 cell surface expression by IL-13 was mediated by the PPARγ signaling pathway. The implication of PPARγ in the transcriptional regulation of Dectin-1 is also confirmed by an *in silico* analysis of the Dectin-1 promoter using Genomatix software. One putative PPARγ responsive element was found in the reverse strand of this promoter. These results highlight that PPARγ is required for the maturation of alternatively activated macrophages. Indeed, we have previously shown that the PPARγ pathway was required *in vitro* and *in vivo* for the induction of the expression of the M2 marker MR (CD206) and CD36 expression during alternative activation of monocytes/macrophages by IL-13 [13],[30],[31]. These results are consistent with the studies of Odegaard and colleagues who showed that the expression of genes preferentially expressed in alternatively activated macrophages such as *Mrc1* (gene of MR CD206) and *Clec7a* (gene of Dectin-1) was decreased by 70–80% in the white adipose tissue of macrophage-specific PPARγ knockout mice [22]. Moreover, a recent study showed that PPARγ activation primed human monocytes into an enhanced M2 phenotype [32]. These authors also reported that thiazolidinediones treatment significantly increased the expression of the M2 marker MR (CD206) in PBMC isolated from patients. In our study, we also show that both 15-ΔPGJ_2_ and rosiglitazone up-regulated Dectin-1 expression through PPARγ. Indeed, in the absence of PPARγ in the murine macrophage cell line RAW 264.7, IL-13 or PPARγ agonists do not induce the increase of Dectin-1 expression, and the effect of the PPARγ agonists or of IL-13 on this expression is restored by the pCMV-PPARγ transfection in these cells. These findings join a paradigm initiated by Huang and coworkers for the regulation of nuclear receptor function by Th2-type cytokines in an alternative pathway of macrophage activation [33].

In this manuscript, we determined the signaling pathway triggered by IL-13 resulting in the increase of Dectin-1 expression. The use of MAFP, a cPLA_2_ inhibitor, blocked the Dectin-1 surface induction by IL-13 and this Dectin-1 over-expression is restored by the addition of 15-ΔPGJ2. Thus, we showed that IL-13 can positively regulate Dectin-1 expression partly by controlling the production of PPARγ endogenous ligands through cPLA_2_ activation. These data are supported by the work of Huang and coworkers who showed that IL-4 leads to the production of PPARγ endogenous ligands and by our confocal microscopy studies illustrating that IL-13 generates 15-ΔPGJ2 production and this nuclear localization in human monocytes and murine macrophages [30],[31],[33].

In summary, we have established that Dectin-1 is essential both to trigger the phagocytosis of non-opsonized *C.albicans* and respiratory burst after yeast challenge during alternative macrophage activation and to control fungal GI infection. We have also demonstrated the major contribution of the MR for the initial recognition of non-opsonized *C.albicans*. These findings suggest that the cooperation between Dectin-1 and the MR is necessary to orchestrate the antifungal response. Moreover, these results underline the importance of the IL-13/PPARγ/C-type lectin receptors axis for the antifungal response in macrophages and in the decrease of colonization of the gastrointestinal tract by *C. albicans*. Indeed, PPARγ ligand strongly enhances the expression of C-type lectin receptors at the surface of macrophages and hence promotes antifungal host defense. These data suggest new therapeutic strategies using PPARγ ligands against fungal infections in immunocompromised hosts and during metabolic diseases, because they increase the innate immune response by enhancing the expression of both the MR and Dectin-1 that are heavily involved in the recognition and elimination of non-opsonized *C.albicans*.

## Materials and Methods

### Ethics statement

This study was carried out in accordance with Approval No. A3155503 and all procedures for animal care and maintenance conformed with the French and European Regulations (Law 87–848 dated 19/10/1987 modified by Decree 2001-464 and Decree 2001-131 relative to European Convention, EEC Directive 86/609 dated 24/11/1986).

### *Candida albicans* strain

The strain of *C. albicans* used throughout these experiments was isolated from a blood culture of a patient in the Toulouse-Rangueil University Hospital. The isolate was identified as *Candida albicans* based on common laboratory criteria and cultured on Sabouraud dextrose agar (SDA) plates containing gentamicin and chloramphenicol. *Candida albicans* was maintained by transfers on SDA plates. Growth from an 18- to 24-h SDA culture of *C. albicans* was suspended in sterile saline.

Fluorescent *C.albicans* was prepared by adding *C.albicans* to fluoroscein isothiocyanate (FITC; Sigma, France) dissolved in sodium carbonate buffer (pH 9.5) at room temperature for 3 h and washed by centrifugation three times in sodium carbonate buffer before storage in aliquots of water at 4°C. The viability of FITC-yeasts was not altered by the protocol of FITC-labeling.

### Reagents

The culture medium was Dulbecco's modified Eagle's medium (DMEM, Gibco Invitrogen Corporation, France) supplemented with glutamine (Gibco Invitrogen Corporation) penicillin, streptomycin (Gibco Invitrogen Corporation), and 10% heat-inactivated fetal calf serum (FCS).

Laminarin (soluble β-glucan from *Laminaria digitata*, Sigma) and mannan (from *S. cerevisiae*, Sigma) were prepared as 10 mg/ml stocks in Hepes-buffered saline solution (HBSS, Gibco Invitrogen Corporation, France), filter sterilized, and stored frozen until use. Solutions used during experiments were made at final concentration of 1.25 mg/mL in DMEM-based culture medium.

For the analysis of binding, phagocytosis of *C.albicans* and ROS production, cultured-macrophages were incubated at 4°C for 20 min with mannan and/or laminarin solution. The medium was removed by washing with cold DMEM until the experiment.

### Macrophage-specific Dectin-1 deficient mice

To generate Dectin-1 floxed (*Dectin-1^L2/L2^*) mice, genomic DNA covering the *Dectin-1* locus was amplified from the 129Sv strain using high fidelity PCR. The resulting DNA fragments were assembled into the targeting vector that after linearization by *NotI* was electroporated into 129Sv ES cells. G418-resistant colonies were selected and analyzed for homologous recombination by PCR and Southern blot hybridization. Positive clones were verified by Southern blot hybridization. Therefore genomic DNA was prepared from ES cells, digested with *XbaI* or *SacI*, electrophoresed and transferred to a positively charged nylon transfer membrane (Amersham Biosciences, Saclay, France). A 0.5 kb DNA fragment (*NotI*–*NheI*) located between exons 6 and 7 (3′ probe) and a 0.5 kb DNA fragment (*NotI*–*SacII*) placed between exons 2 and 3 (5′ probe) were used as probes. The karyotype was verified and several correctly targeted ES cell clones were injected into blastocysts from C57BL/6J mice. These blastocysts were transferred into pseudopregnant females, resulting in chimeric offspring that were mated with female C57BL/6J mice that express the Flp recombinase under the control of the ubiquitous CMV promoter. The offspring that transmitted the mutated allele, in which the selection marker was excised and that had lost the Flp transgene (*Dectin-1* ^+/L2^ mice), were then selected and used for systematic backcrossing with C57BL/6J mice to generate congenic *Dectin-1* floxed mouse lines. A PCR genotyping strategy was subsequently used to identify *Dectin-1^+/+^*, ^+/L2^, and ^L2/L2^ mice. To generate phagocyte-specific mutant (LysM-*Dectin-1* ^−/−^) mice, *Dectin-1^L2/L2^* mice were mated with LysM-Cre C57BL/6J mice in which the Cre recombinase was expressed under the control of the phagocyte-selective lysozyme promoter [34]. LysM-Cre/*Dectin-1^L2/+^* mice, heterozygous for the floxed *Dectin-1* allele, were selected and subsequently inter-crossed to generate pre-mutant LysM-Cre/*Dectin-1^L2/L2^* mice. At least two more rounds of breeding were required to generate age- and sex-matched mice for the experimental cohorts.

### Primary cell culture

Murine resident peritoneal cells were harvested from female wild-type or macrophage-specific Dectin-1 deficient mice. Briefly, cells were obtained by injection into the peritoneal cavity of sterile HBSS. The collected cells were centrifuged, and the cell pellet was suspended in culture medium as described in “Reagents” section. Cells were allowed to adhere over 2 h at 37°C with 5% CO2 atmosphere in 24- or 96-well culture plates. Nonadherent cells were removed by washing with phosphate-buffered saline (PBS) (Gibco Invitrogen Corporation), and the remaining adherent cells were stimulated as described below.

### Stimulation assays

Peritoneal macrophages were stimulated by rosiglitazone (5 µM), 15d-PGJ2 (1 µM), MCC555 (5 µM), GW1929 (1 µM) (Cayman Chemical, USA), or IL-13 (50 ng/mL) (Sanofi-Synthelabo, France). In some experiments, macrophages were incubated with the specific inhibitors of PPARγ, GW9662 (5 µM) and T0070907 (2 µM) (Cayman Chemical, USA) or of cPLA2, MAFP (10 µM, 20 µM) (Cayman Chemical, USA), 10 min before the addition of PPARγ ligands or IL-13. Macrophages were incubated for 20 h for binding, phagocytosis, and ROS assays and quantification of surface expressed markers; cells were cultured for 4 h for transcript quantifications.

### Binding assay

For the analysis of the binding of *C.albicans*, 5.10^5^ cultured-macrophages were incubated at 4°C for 20 min with mannan solution. The medium was removed by washing with cold DMEM, and peritoneal macrophages were subsequently challenged with six FITC-labeled yeasts per macrophage and binding was performed at 4°C. Binding was stopped after 20 min by washing the macrophages with ice-cold PBS. Macrophage monolayers were incubed with ice-cold PBS and gently scraped.The amount of *C.albicans* binding to the macrophages was determined using FACS based approach. The fluorescence was quantified on a Becton Dickinson FACScan using CellQuestPro software and used as indicator of the binding efficiency.

### Phagocytosis assay

For analysis of phagocytosis of *C.albicans*, 5.10^5^ cultured-macrophages were incubated at 4°C for 20 min with mannan and/or laminarin solution. The medium was removed by washing with cold DMEM, and the peritoneal macrophages subsequently challenged with six FITC-labeled yeasts per macrophage and phagocytosis was initiated at 37°C in an atmosphere of 5% CO2. Phagocytosis was stopped after 60 min by washing the macrophages with ice-cold PBS. Macrophage monolayers were incubed with ice-cold PBS-EDTA (5 mM) and gently scraped. The amount of *C.albicans* engulfed by macrophages was determined using FACS based approach. The distinction between internalized yeast cells and those attached to macrophage surface was done *via* quenching the FITC-fluorescence with trypan blue. The remaining fluorescence was quantified on a Becton Dickinson FACScan using CellQuestPro software and used as indicator of the phagocytosis efficiency.

### Assay for the production of ROS

The macrophages were plated in 96-well Falcon plates (2.10^5^ macrophages/well). The oxygen-dependent respiratory burst of macrophages was measured by chemiluminescence (CL) in the presence of 5-amino-2,3-dihydro-1,4-phthalazinedione (luminol) using a thermostatically (37°C) controlled luminometer (Wallac 1420 Victor2, Finland). The generation of CL was monitored continuously for 1 hr after incubation of the cells with luminol (66 µM) and after *Candida albicans* challenge at a yeast-to-macrophage ratio of 3∶1 or non-opsonized zymozan (ZNO) at final concentration of 2 µg/mL. Statistical analysis was performed using the area under the curve expressed in counts x seconds.

### Flow cytometry

After 20 h of culture, the culture medium was removed and macrophage monolayers were incubated with ice-cold PBS-EDTA (5 mM) and gently scraped. After washing by centrifugation, the surface Dectin-1 or CD36 expressions were detected respectively using FITC-Dectin-1 mAb (Serotec, Düsseldorf, Germany) or PE-CD36 mAb (Tebu-Santa Cruz) and compared with an irrelevant appropriate isotype control. To characterize Cre (0/Tg) macrophages, the analysis was performed on non adherent cells. The labeled mAbs anti-F4/80-PE-Cy5, anti-CD11b-Alexa 647, and anti-TLR2-alexa 488 were obtained from Serotec. mAb anti-SIGNR1 and anti-TLR4 were obtained from eBioscience, and anti-CD14 was obtained from (BD PharMingen). To evaluate the MR surface expression, we have used a MR-specific ligand conjugated to FITC; macrophages were incubated with FITC-labeled mannosylated bovine serum albumin. A population of 5000 cells was analyzed for each data point. All analyses were done in a Becton Dickinson FACScan using CellQuestPro software.

### Reverse transcription and real-time PCR

Total RNA was prepared with RNeasy® Mini Kit columns (QIAGEN) using the manufacturer's protocols. The synthesis of cDNA was completed with QuantiTect® Reverse Transcription (QIAGEN) according to the manufacturer's recommendations and primed with hexamers. Quantitative real-time PCR was performed on a LightCycler system (Roche Diagnostics) using QuantiFastTM SYBR® Green PCR (QIAGEN). Ten microliters of reaction mixture were incubated; the amplifications were performed for 40 cycles (10 s at 95°C and 60 s at 60°C) for Dectin-1 and β-actin. The primers (at a final concentration of 10 mM) were designed with the software Primer Express (Applied Biosystems, Foster City, CA). Sequences were as follows: (sens) 5′-TGG AAT CCT GTG GCA TCC ATG AAA-3′; (antisens) 5′-TAA AAC GCA GCT CAG TAA CAG TCC G 3′ for β-actine, and (sens) 5′-CAT CGT CTC ACC GTA TTA ATG CAT-3′ (antisens) 5′-CCC AGA ACC ATG GCC CTT-3′ for Dectine-1.

Real-time PCR data are represented as Ct values, where Ct was defined as the crossing threshold of PCR by the Light-Cycler® System. For calculating relative quantification of β-GR mRNA expression, we have used the following procedure.

ΔCt_β-GR_ = Ct_Sample_−Ct_Vehicle_.ΔCt_β-actin_ = Ct_Sample_−Ct_Vehicle_. Then, ΔΔCt represented the difference between ΔCt_β-actin_ and ΔCt _β-GR_ calculated by the formula ΔΔCt = ΔCt_β-actin_−ΔCt_β-GR_. Finally, the N-fold differential expression of β-GR mRNA samples compared to the vehicle was expressed as 2^ΔΔCt^.

Each experiment was performed independently at least three times and the results of one representative experiment are shown.

### Transfection assay

For the plasmid transfection, the macrophage murine cell line RAW264.7 was maintained in an exponential growth phase by subsequent splitting in DMEM complemented with 10% of FCS. The day prior to transfection, cells were splited in 24-well dishes. Then the complete medium was replaced by DMEM without any serum and the cells were transiently transfected for 18 h at 60–80% of confluence with Fugen 6 (Roche, Switzerland). The ratio of DNA/Fugen was 1∶2 with 1 µg of DNA. On the day of stimulation, the medium was discarded and fresh complete medium was added with stimulations as indicated in the figures. The pCMV-luc (CEA, France) served as a control. The pCMV-mPPARγ, a gift from Ron Evans (The Salk Institute, San Diego, CA), was encoded for the mouse nuclear receptor PPARγ.

For the siRNA transfection, siRNA control and siRNA to knockdown PPARγ (sc-29456) were transfected into murine peritoneal macrophages with the siRNATransfection Reagent in the siRNA Transfection medium as described in manufacturer's protocol (Santa Cruz biotechnology, Inc.)

### Cytokine titration

For *in vitro* cytokine expression, peritoneal macrophages were added to 96-well plates (2.10^5^ macrophages/well) and then stimulated with heat-killed *C.albicans* at a yeast-to-macrophage ratio of 3∶1 for 24 h, or with non-opsonized zymosan at a final concentration of 2 µg/mL. or with LPS at a final concentration of 100 ng/mL. Supernatants were recovered and frozen at −70°C before analysis. The production of TNFα in the cell supernatants was determined with a commercially available OptiEIA kit (BD Biosciences) according to the manufacturer's instructions.

### Arachidonic acid mobilization study

Murine peritoneal macrophages were prelabeled with [^3^H]arachidonic acid. Briefly, adherent murine peritoneal macrophages (5×10^5^ per well in 24-well plates) were cultured for 18 hours at 37°C under an atmosphere of 5% CO2, in DMEM (0.5 mL) containing 1% FCS and 1 µCi/mL [^3^H]arachidonic acid as previously described [35]. After 18 h, the culture medium was removed and pre-labeled macrophages were washed three times with 0.5 mL DMEM containing 1% FCS; after, the cells were treated or not with 100 nM of phorbol 12-myristate 13-acetate (PMA) or *C.albicans* at a ratio of 3∶1 (yeast∶macrophage) for 2 h. The [^3^H]arachidonic acid metabolites released into the culture medium by stimulated or unstimulated macrophages were quantified by measurement of the radioactivity by beta liquid scintillation counting using a 1217 Wallac Rackbeta LKB 1217.

### Quantification of *Candida albicans* in the gastrointestinal tract and visceral organs: Light Cycler-based PCR assay

For *C.albicans* DNA extraction, 250 µL of each tissue homogenate was prepared with the High Pure PCR Template preparation kit (ROCHE) using the manufacturer's protocols.

The Light Cycler PCR and detection system (Roche Diagnostics, Mannheim, Germany) was used for amplification and online quantification as previously described [13].

### Mice infection

All animal experimentation was conducted in accordance with accepted standards of humane animal care. The model of gastrointestinal candidiasis was established in 8-week-old female control or macrophage-specific Dectin-1 deficient mice. Mice were given 0.3 mL of the yeast suspension by the oral route (5.10^6^ or 5.10^7^ *C.albicans* CFU per mouse).

### Treatment groups

Therapeutic studies were performed on separate groups of 6 mice each infected with *C. albicans*. Mice received the treatment in 500 µl of NaCl 0.9% by the intraperitoneal route. The final DMSO concentration was lower than 0.1% (v/v). Mice were treated with rosiglitazone (Cayman) one day prior to infection, the day of infection and then every two days with a dose of 2.8 µg/g of mouse.

No colonized animals died during the course of the study. On day 5, all mice were euthanized using CO_2_ asphyxia and the peritoneal cells harvested. Macrophages of infected animals were used to investigate phagocytosis and ROS production and to evaluate the surface expression of the MR. Previous data shown that *Candida* infection had no effect on effector macrophage functions (phagocytosis and ROS production).

In parallel, standardized samples of stomach and cecum were aseptically removed and homogenized in 400 µL of sterile-normal saline using tissue-lyser beads (MP biomedical). Fungal burdens of the tissues are shown as log yeasts per gram of tissue after quantification of *C.albicans* by RT-PCR.

### Statistical analysis

For each experiment, the data were subjected to one-way analysis of variance followed by the means multiple comparison method of Bonferroni-Dunnett. p<0.05 was considered as the level of statistical significance.
